# Human menstrual cycle variation in subcortical functional brain connectivity: a multimodal analysis approach

**DOI:** 10.1007/s00429-019-02019-z

**Published:** 2020-01-01

**Authors:** Esmeralda Hidalgo-Lopez, Karsten Mueller, TiAnni Harris, Markus Aichhorn, Julia Sacher, Belinda Pletzer

**Affiliations:** 1 Department of Psychology and Centre for Cognitive Neuroscience, University of Salzburg, Hellbrunnerstr. 34, 5020 Salzburg, Austria; 2 Methods and Development Group Nuclear Magnetic Resonance, Max Planck Institute for Human Cognitive and Brain Sciences, Stephanstrasse 1a, 04103 Leipzig, Germany; 3 Research Group EGG (Emotions and neuroimaGinG)-Laboratory, Max Planck Institute for Human Cognitive and Brain Sciences, Stephanstrasse 1a, 04103 Leipzig, Germany; 4 Clinic for Cognitive Neurology, University Hospital Leipzig, Liebigstrasse 16, 04103 Leipzig, Germany

**Keywords:** Resting state, Menstrual cycle, Intrinsic connectivity networks (ICN), Eigenvector centrality mapping (ECM), Amplitude of low-frequency fluctuations (ALFF), Seed-based connectivity

## Abstract

Increasing evidence suggests that endogenous sex steroid changes affect human brain functional connectivity, which could be obtained by resting-state fMRI (RS-fMRI). Nevertheless, RS studies on the menstrual cycle (MC) are underrepresented and yield inconsistent results. We attribute these inconsistencies to the use of various methods in exploratory approaches and small sample sizes. Hormonal fluctuations along the MC likely elicit subtle changes that, however, may still have profound impact on network dynamics when affecting key brain nodes. To address these issues, we propose a ROI-based multimodal analysis approach focusing on areas of high functional relevance to adequately capture these changes. To that end, sixty naturally cycling women underwent RS-fMRI in three different cycle phases and we performed the following analyses: (1) group-independent component analyses to identify intrinsic connectivity networks, (2) eigenvector centrality (EC) as a measure of centrality in the global connectivity hierarchy, (3) amplitude of low-frequency fluctuations (ALFF) as a measure of oscillatory activity and (4) seed-based analyses to investigate functional connectivity from the ROIs. For (2)–(4), we applied a hypothesis-driven ROI approach in the hippocampus, caudate and putamen. In the luteal phase, we found (1) decreased intrinsic connectivity of the right angular gyrus with the default mode network, (2) heightened EC for the hippocampus, and (3) increased ALFF for the caudate. Furthermore, we observed (4) stronger putamen–thalamic connectivity during the luteal phase and stronger fronto-striatal connectivity during the pre-ovulatory phase. This hormonal modulation of connectivity dynamics may underlie behavioural, emotional and sensorimotor changes along the MC.

**Electronic supplementary material:**

The online version of this article (10.1007/s00429-019-02019-z) contains supplementary material, which is available to authorized users.

## Introduction

Sex hormones are well known for their structural and functional implications in the central nervous system (McEwen and Milner 2017). Several lines of evidence suggest that subtle fluctuations of endogenous sex steroids along the menstrual cycle impact brain structure and functional brain organization. In animal research, there is strong evidence for estrogen-dependent synaptic remodeling in the hippocampus (Woolley and McEwen 1993; Yankova et al. 2001; Qiu et al. 2013) and prefrontal cortex (Khan et al. 2013). Likewise, progesterone has also been shown to increase dendritic spine number and density in cortical neuron cultures (Sanchez et al. 2013; Acharya et al. 2017). Although this suggests similar mechanism of action for both sex hormones, animal data strongly indicate that the effects are context dependent and progesterone might facilitate some of these estrogenic actions, while modulating or opposing others (McEwen and Milner 2017). Therefore, to appropriately assess their combined effects along the menstrual cycle, longitudinal designs are required. In line with the animal literature, human studies also lend support to a trophic effect of estradiol on the hippocampus (Eberling et al. 2004). For instance, hippocampal gray matter (GM) volumes increase during the pre-ovulatory phase, when estradiol levels peak (Protopopescu et al. 2008; Lisofsky et al. 2015; Barth et al. 2016; Pletzer et al. 2018). Furthermore, GM volume in the basal ganglia increase with higher progesterone levels during the mid-luteal cycle phase (Protopopescu et al. 2008; Pletzer et al. 2018). Despite the complex relation between structural and functional connectivity (Pessoa 2014), given the acute trophic effects on dendritic spine density and consequent synapse formation, these effects would also be expected to reflect on brain function and connectivity (Kitamura et al. 2018). Indeed, higher brain activity has been observed in hippocampus before ovulation and in basal ganglia during the luteal phase (Pletzer et al. 2019). Furthermore, both at rest and during different tasks, increased functional cortico-subcortical connectivity has been reported during the higher hormone phases (Peper et al. 2011; Arélin et al. 2015). The fact that the majority of these changes occur irrespective of the task, suggests a menstrual cycle modulation of common underlying functional networks.

Resting-state functional MRI (RS-fMRI) offers the most valuable resource to investigate the neural functional network without the interference of a task (Cole et al. 2010). Similar to other methods, RS-fMRI also has its pitfalls, such as the absence of reference to establish the strength of the BOLD signal or not being a direct proxy for anatomic connectivity (Buckner et al. 2013). Nevertheless, it has proven to be a powerful tool to outline human functional connectivity (Greicius et al. 2009). To this end, different methodological approaches have been used for its assessment, leading to different interpretations (see Table 1 for an overview of methods; for review, see Margulies et al. 2010). In menstrual cycle research, only few studies have applied RS-fMRI to investigate changes in intrinsic functional connectivity along the phases (see Table 2 for an overview of studies). The majority of these studies focused on intrinsic connectivity networks (ICNs), derived by independent component analyses (ICA) (Hjelmervik et al. 2014; Petersen et al. 2014; De Bondt et al. 2015; Pletzer et al. 2016). The ICNs are functional sets of brain regions consistent across subjects with temporally correlated activity during rest (Damoiseaux et al. 2006; Fox et al. 2007), suggesting that they relate to underlying neural activity and reflect brain coupling dynamics (Stern 2002; Beckmann et al. 2005; Fox and Raichle 2007; Britz et al. 2010; Thomas Yeo et al. 2011). Most menstrual cycle studies have focused on a few selected networks (default mode network—DMN, executive control network—ECN), arriving at mixed results (Table 2). The DMN has been more consistently reported to change across the cycle, increasing its connectivity with the left middle frontal gyrus during menses (Weis et al. 2019) and decreasing its connectivity with the left angular gyrus during the luteal phase (Petersen et al. 2014). Exploring menstrual cycle changes in other ICNs, Pletzer et al. (2016) found for instance that during the luteal cycle phase connectivity of the basal ganglia increased with the right fronto-parietal attention network, while it simultaneously decreased with subcortical networks related to emotion and automatic processes.

**Table 1:** Overview of methodological approaches and main results in the present study

Method	Measure	Interpretation	References	Advantages	Limitations	Main results	
						Pre-O	L
Group ICA	Multivariate method that decomposes spatially independent but temporally coherent networks	Distinct ICNs correspondent to functionally relevant networks	(Mckeown et al. 1998; Calhoun et al. 2001)	- Reliable and consistent functional connectivity patterns - Data driven, no assumptions about ROIs -Noise extracted as component/s	- Number of components unknown - No reproducibility due to random assumption at the beginning of iterative process - Networks identified a posteriori with spatial templates	n.s	↓DMN-rAng related to ↓E and ↑P^2^
ECM	Graph-based method that weights nodes based on their correlations with other nodes within the network	Functional relevance and hierarchy of a node within the brain network	(Bonacich 1972; Lohmann et al. 2010)	- Describe and characterize intrinsic properties of the brain network - Parameter-free, not depending on prior assumptions - More sensitive to subcortical regions	- Analytic approaches are computationally difficult - Ambiguous measure difficult to relate to specific cognitive function	n.s	↑Hipp^2^
ALFF	Strength of BOLD-signal fluctuation by the total power within a range of 0.01–0.08 Hz	Spontaneous local oscillatory activity	(Zang et al. 2007)	- Characterize spontaneous local brain activity -Conceptually straightforward and easily implemented	- Sensitive to physiological, neural and artifactual factors	n.s	↑Cd related to ↓E and ↑P^1,2^
Seed based connectivity	Temporal correlation of a neurophysiological index from a selected “seed region” and all other voxels in the brain	Functional connectivity patterns	(Biswal et al. 1995)	- Straightforward analytical approach and comprehensible results	- Seed regions need to be selected a priori	↑rCd-rMFG^1^	↑lPut-rTh related to ↑E^1^

*ICA* independent component analyses, *ECM* eigenvector centrality mapping, *ALFF* amplitude of low-frequency fluctuations, *Pre-O* pre-ovulatory, *L* luteal, *E* estradiol, *P* progesterone, *n.s.* not significant, *ICN* intrinsic connectivity networks, *DMN* default mode network, *Ang* angular gyrus, *Hipp* hippocampus, *Cd* Caudate, *Put* putamen, *MFG* middle frontal gyrus, *Th* thalamus, *r* prefix: right, *l* prefix: left

^1^Compared to menses

^2^Compared to pre-ovulatory

**Table 2:** Overview of menstrual cycle literature in RS fMRI

Method	References	*N*	Design	Whole brain/ ICN/ROI	Phases considered	Cycle comparisons		Hormone relations	
						Pre-O	L	E	P
Group ICA	Hjelmervik et al. 2014	16	Longitudinal	FPN	M, Pre-O, L	n.s		n.s	
	Petersen et al. 2014	20 M 25 L	Cross-sectional	DMN and ECN	M, L	Not considered	↓DMN-lAng^1^ ↓ECN-ACC^1^	n.s	
	De Bondt et al. 2015	18	Longitudinal	DMN and FPN	M, Pre-O, L	n.s		↑Precuneus	n.s
	Pletzer et al. 2016	18	Longitudinal	DMN, FPN, ECN, MLN	M, Pre-O, L	↑DMN-lTemp^1^ ↓MLN-BG^1^	↑DMN-Cuneus^1,2^ ↑rFPN-mPFC/BG^1,2^ ↓lFPN-rSMC/ rOperculum1,2 ↓MLN-Precuneus/BG^1,2^	Not considered	
	Weis et al. 2019	19	Longitudinal	DMN	M, Pre-O, L	↓DMN-ldlPFC^1^	n.s	Not considered	
	Syan et al. 2017	25	Longitudinal	DMN, FPN, MLN	M, L	Not considered	n.s	Not considered	
ECM	Arélin et al. 2015	1	Single subject 32 scans	Whole brain	Four entire MC	Not considered		n.s	↑ dlPFC ↑ SMC
ALFF	Not considered								
Seed based connectivity	Syan et al. 2017	25	Longitudinal	PCC, dlPFC, aI, Amyg, V1, SMC	M, L	Not considered	n.s	↑ Amyg-SMC	↓dlPFC-SMC
	Engman et al. 2018	18	Longitudinal	Amyg and dACC	M, L	Not considered	↑Amyg-dlPFC/SMC/CB^1^ ↑dACC-dlPFC/Temp/SMC^1^	Not considered	
	Arélin et al. 2015	1	Single subject 32 scans	dlPFC and SMC	Four entire MC	Not considered		n.s	↑dlPFC-Hipp ↑SMC-Hipp

*ICA* independent component analyses, *ECM* eigenvector centrality mapping, *ALFF* amplitude of low-frequency fluctuations, *M* menses/early follicular, *Pre-O* pre-ovulatory, *L* luteal, E:estradiol, *P* progesterone, *n.s.* not significant, *MC* menstrual cycle, *ICN* intrinsic connectivity network, *ROI* region of interest, *FPN* fronto-parietal network, *DMN* default mode network, *ECN* executive control network, *MLN* mesolimbic network, *Ang* angular gyrus, *d/ACC* dorsal/anterior cingulate cortex, *mPFC* medial prefrontal cortex, *BG* basal ganglia, *SMC* sensoriomotor cortices, *PCC* posterior cingulate cortex, *dlPFC* dorsolateral prefrontal cortex, *aI* anterior insula, *Amyg* amygdala, *CB* cerebellum V1: primary visual cortex, *Temp* temporal gyrus, *Hipp* hippocampus, *r* prefix: right, *l* prefix: left. For group ICA analyses, only results in DMN, FPN, ECN, and MLN are summarized

^1^Compared to menses

^2^Compared to pre-ovulatory

Indeed, the basal ganglia have been described as a hub of the so-called rich club, a phenomenon where key nodes in overall brain network are more densely connected among themselves than with others, creating higher order networking structure (van den Heuvel and Sporns 2011). This higher functional relevance corresponds to a key role in whole-brain communication and integration of different functional brain modules. Functional and structural results suggest the basal ganglia as a functional connector between networks (Alexander and Crutcher 1990), acting as a switch from one network to another during different phases of the menstrual cycle (Pletzer et al. 2016). If sex hormones modulate RS connectivity along the menstrual cycle via rich club hubs, the question arises whether they also affect the hierarchical structure of RS brain connectivity. One measure to address such hierarchical patterns in global network connectivity is eigenvector centrality (EC) (Lohmann et al. 2010; Sato et al. 2014). EC is a graph-based measure that takes into account the centrality of all adjacent nodes (Bonacich 1972), using an algorithm similar to Google’s PageRank (Brin and Page 1998). The higher the centrality of the connected nodes, the higher the EC of the node itself. Accordingly, in a network, the centrality of a node assesses its functional importance (Koschützki et al. 2005). It has been suggested that EC mapping is more sensitive to subcortical regions, possibly, because they are not that widely connected but to key hubs (Zuo et al. 2012). So far, only one study has addressed whether EC changes along the menstrual cycle (Arelin et al. 2015). In a longitudinal single-subject study, they found higher progesterone levels to relate to a higher EC in dorsolateral prefrontal (DLPFC) and sensorimotor cortex. A subsequent seed-based analysis from these regions of interest (ROIs) showed changes in connectivity to bilateral hippocampus related to progesterone levels. Relatedly, the hippocampus has been also identified as a rich club hub and robust evidence suggests it to be among the brain regions most susceptible of modulation by the menstrual cycle. Other seed-based studies focused on the amygdala and dorsal ACC (Engman et al. 2016, 2018) finding higher functional connectivity of these areas during cycle phases with higher hormonal levels. However, until now, all these attempts to unravel menstrual cycle-dependent changes in RS connectivity have arrived at quite different conclusions and no clear picture emerges. One reason for these mixed results lies in the number of different methods that have been used on separate samples. A second reason is that hormonal fluctuations along the menstrual cycle are short-lived and likely elicit only subtle effects, which do not consistently show up in exploratory whole-brain analysis in small samples. Nevertheless, such subtle changes may have profound impact on overall network dynamics if they affect key nodes of high functional relevance, e.g., rich club hubs (Heuvel and Sporns 2011).

One way to solve these issues is a multimodal analysis approach in the same large-scale longitudinal dataset using hypothesis-driven region of interest (ROI) selection (Poldrack 2007). Converging findings in human and animal studies provide strong evidence that the basal ganglia and hippocampus are both of high functional significance (Heuvel and Sporns 2011) and potential targets for sex hormone effects (Yankova et al. 2001; McEwen and Milner 2007; Protopopescu et al. 2008; Arélin et al. 2015; Lisofsky et al. 2015; Pletzer et al. 2016, 2018). Accordingly, we aim to map influences of ovarian hormones along the menstrual cycle on these areas, by applying different RS methods to the largest, longitudinal human neuroimaging dataset across the menstrual cycle to date. One method that has not been applied in the menstrual cycle literature so far is the quantification of amplitude of low-frequency fluctuations (ALFF), which provides insight into how strongly the BOLD-signal fluctuates (Zang et al. 2007). The ALFF has been proposed as an index directly reflecting the intensity of spontaneous local neural activity and sensitive mostly to signal from gray matter networks. This measure is often used as a biomarker of altered resting brain activity in clinical populations or as a reflection of a more general effect on the BOLD response (Zang et al. 2007; Qi et al. 2012). This is of particular relevance with regards to sex hormonal effects, since it has been demonstrated that there is an increase in overall cerebral blood flow during the pre-ovulatory phase (Peltonen et al. 2016), but not in resting cerebral perfusion (Ances and Detre 2003).

To investigate menstrual cycle-dependent changes in the RS brain and facilitate comparison across previously published studies, (1) we first performed a group ICA and investigated menstrual cycle-dependent changes in the emerging ICNs. We then performed hypothesis-driven ROI analyses for (a) hippocampus, (b) caudate and (c) putamen using the (2) EC method as a measure of centrality in the global connectivity hierarchy, the (3) ALFF method, as a measure of oscillatory activity, and (4) seed-based analyses to investigate functional connectivity. We predict an increased activation and functional connectivity associated to phases of higher hormonal levels. Specifically, we hypothesize an increase in hippocampal EC and ALFF during the pre-ovulatory cycle phase compared to menses, as well as an increase in the EC and ALFF of the basal ganglia in the luteal phase compared to menses. As secondary analyses, we also explored the whole-brain level for EC and ALFF.

## Methods

### Participants

Seventy-eight healthy young women participated in 1 of 2 functional imaging studies. Main inclusion criteria were an age range between 18 and 35 years, and having a regular menstrual cycle, defined as ranging between 21 and 35 days, with a variability of cycle length between individual cycles of less than 7 days (Fehring et al. 2006). Exclusion criteria included use of hormonal contraceptives within the previous 6 months, neurological, psychiatric or endocrine disorders, and any medication intake. Due to inconsistencies between self-reported cycle phase and hormone levels, 18 women were excluded, resulting in a total sample of 60 healthy young women (see Table 3 for demographics). All participants had achieved general qualification for university entrance and their IQ was measured on the Raven’s APM Screening as implemented in the Vienna Test System (WTS). All participants gave their informed written consent to participate in the study. All methods conform to the Code of Ethics by the World Medical Association (Declaration of Helsinki). Both studies were approved by the University of Salzburg’s ethics committee.

**Table 3:** Demographic data and hormone levels during each cycle phase

Sample (*n* = 60)	Age (years)	APM (IQ)	Cycle length (days)	First scanning session	Cycle day of assessment (days)	Estradiol (pg/ml)	Progesterone (pg/ml)
Menses	25.40 ± 0.55	110.55 ± 1.19	28.28 ± 0.30	19	3.72 ± 0.19	0.84 ± 0.06**	68.77 ± 5.73
Pre-ovulatory				21	12.08 ± 0.31	1.17 ± 0.08***	91.17 ± 8.69
Luteal				20	21.37 ± 0.46	0.99 ± 0.06**	207.52 ± 18.67***

Values are presented as mean ± standard error of the mean (M ± SEM) for the final sample of *n* = 60

For hormone levels, *corresponds to *p* < 0.05, **corresponds to *p* < 0.01, and ***corresponds to *p* < 0.001

### Procedure

In both studies, all participants underwent three scanning sessions, time-locked to their menses, pre-ovulatory phase or mid-luteal phase. For cycle phase estimation, cycle length was estimated based on participants’ self-reports of the onsets of their last three menstrual periods. Based on cycle length, the expected onset of the next menses was calculated and ovulation was estimated to fall 14 days before the onset of next menses. Menses sessions were scheduled 2–6 days after the onset of current menses; pre-ovulatory sessions were scheduled 2–3 days before the expected date of ovulation and complemented by commercially available urinary ovulation tests (Pregnafix^®^). Mid-luteal sessions were scheduled in a window between 3 days after ovulation and 3 days before the expected onset of next menses (see Table 3). Participants had to confirm the onset of next menses in retrospect.

### Hormone analysis

During each scanning session, two–four saliva samples were collected from participants, one–two before and one–two after entering the scanner. Until hormone assessment, saliva samples were stored at − 20 °C centrifuged at 3000 rpm for 15 min and 10 min, respectively. To obtain an averaged value over the scanning session, samples were pooled prior to analyses. Estradiol and progesterone were assessed using the Salimetrics High Sensitivity salivary Estradiol assay and the DeMediTec Progesterone free in saliva ELISAs, respectively.

### fMRI data acquisition

Functional images as well as high-resolution structural images were acquired on Siemens Magnetom TIM Trio 3 T scanner (Siemens Healthcare). In both studies, an RS scan of about 9 min duration was performed as the second scan of the protocol following a fieldmap. We used a *T*2*-weighted gradient echo planar (EPI) sequence with 36 transversal slices oriented parallel to the AC–PC line (whole-brain coverage, TE = 30 ms, TR = 2250 ms, flip angle 70°, slice thickness 3.0 mm, matrix 192 × 192, FOV 192 mm, in-plane resolution 2.6 × 2.6 mm). Participants were instructed to close their eyes, relax and let their mind flow. A high-resolution structural scan was performed as fourth scan of the sequence. We used a T1-weighted 3D MPRAGE sequence (160 sagital slices, slice thickness = 1 mm, TE 291 ms, TR 2300 ms, TI delay 900 ms, FA 9°, FOV 256 × 256 mm).

### fMRI data analyses

As a first step of the analysis pipeline, functional images were despiked using 3D-despiking as implemented in AFNI (afni.nimh.nih.gov). The despiked images were then pre-processed using SPM12 standard procedures and templates including (1) realignment and unwarping of the functional images using the fieldmap, (2) segmentation of the structural images using CAT12, (3) co-registration of the functional images to the structural images, (4) normalization of functional images using the normalization parameters as estimated by CAT12, (5) spatial smoothing using a 6 mm kernel. Additionally, for the ECM and ALFF, the resulting images were then subjected to the ICA-AROMA algorithm implemented in fsl and non-aggressive removal of artifactual components was chosen (Pruim et al. 2015). Finally, cerebrospinal fluid (CSF) and white matter (WM) masks were created to demean and regress the CSF, WM values. A high-pass filter of 0.01 Hz, and a linear detrend was applied, followed by the addition of previously removed mean. To restrict the EC and ALFF analyses to GM regions, the images were masked with a binary GM image.

#### Group ICA

After the common preprocessing steps and as previously described in Pletzer et al. (2016), ICNs were identified by a group level ICA using the ICASSO algorithm implemented in the GIFT toolbox (mialab.mrn.org/software/gift/), version 2.0. Group ICA was performed to extract temporally coherent functional networks which are spatially non-overlapping (Calhoun et al. 2008). This approach temporally concatenates each single-subject data and aggregates spatial ICA analysis. Regular back reconstruction for single-subject maps and time courses, intensity normalization, and expectation maximization were applied (Calhoun et al. 2008). Back-reconstructed individual subject maps were scaled to *z*-scores for the second-level analysis to compare the strength of connectivity within each network voxel-wise between the three cycle phases. A fixed number of 20 components were extracted and to ensure functionally relevant networks, we use the RS network taxonomy described by Laird et al. (2011) and assessed our ICNs via spatial correlations in GIFT (see Table 1 in Supplementary material). Three of the 20 components (2, 18 and 20) showed predominant activation in peripheral areas, the cerebrospinal fluid, and white matter and thus were discarded as artefactual.

For the second-level analyses, each of the components was transformed into a NifTI image file using the MARSeille Boîte À Région d’Intérêt toolbox (MarsBaR; https://marsbar.sourceforge.net; Brett et al. 2002), and used as an explicit mask. Finally, the component images for each subject were compared between cycle phases using a flexible factorial design in SPM12.

#### ECM

For each participant and session, EC mapping analysis (Lohmann et al. 2010) was computed using the add option of the LIPSIA software package (Lohmann et al. 2001), as previously described in Arélin et al. 2015 and Hove et al. 2016. With this method, each voxel included in the analysis has a centrality value assigned which indicates its hierarchically weighted connectivity degree with the rest of the voxels.

#### ALFF

The ALFF is defined as the average square root of each frequencies power within a range of 0.01–0.08 Hz as obtained from the power spectrum after a Fourier transform. Therefore, a bandpass filter of 0.01–0.08 Hz was applied to remove effects of very-low-frequency drift and high-frequency noise as caused, e.g. by respiratory and heart rhythms (Zang et al. 2007) and ALFF maps were calculated using the DPABI toolbox (Yan et al. 2016).

#### ROI analyses

For the ECM and ALFF approaches, subsequent region of interest (ROI) analyses were performed. Eigenvalues were extracted from one-sample *t* tests over all subjects and sessions restricted to bilateral hippocampus, caudate and putamen. To define the six ROIs, we used masks based on the Brodmann Areas as implemented in the Wake Forest University (WFU) Pickatlas toolbox (Maldjian et al. 2003). We then entered these values as dependent variables into a linear mixed model as further detailed in the statistical analysis section. To further explore whole-brain effects of the menstrual cycle, EC and ALFF maps were compared between cycle phases using a flexible factorial design in SPM12. We used an extent threshold of *k* = 30 voxels, an uncorrected primary threshold of *p* < 0.001 and a secondary cluster-level FWE-corrected threshold of *p* < 0.05 (indicated as pFWE).

#### Seed-based connectivity analyses

To assess the connectivity of each of the ROIs to the rest of the brain, seed-to-voxel connectivity maps were estimated for each subject and session using the CONN-toolbox standard procedures and templates (Whitfield-Gabrieli and Nieto-Castanon 2012). The six movement parameters as well as 5 WM and CSF components were used as regressors during the denoising step. A band-pass filter of 0.008–0.09 Hz was applied. One-sample *t* tests were used to evaluate the overall connectivity pattern of each of the ROIs at the group level. Connectivity maps for each of the ROIs were then compared between cycle phases using a flexible factorial design in SPM12. We used an extent threshold of *k* = 30 voxels, an uncorrected primary threshold of *p* < 0.001 and a secondary cluster-level FWE-corrected threshold of *p* < 0.05 (indicated as *p*_FWE_).

### Statistical analyses

Statistical analyses were performed in R 3.4.0 using linear mixed effects models. All models included participant number (PNr) as random factor, and session as control variable for repeated measurements. Session did not affect neuroimaging parameters, and it is, therefore, not reported further (all |*b*|< 0.08, all SEb < 0.10, all |*t*|< 1.20, all *p* > 0.05). To compare the hormone levels between cycle phases, cycle phase was entered as a fixed effect (e.g., hormone ~ 1|PNr + session + cycle). To explore the menstrual cycle effects on the EC and ALFF values from the ROIs, the eigenvalues were entered as dependent variable, and cycle phase and hemisphere were entered as fixed effects (e.g., eigenvariate ~ 1|PNr + session + cycle × hemisphere). In all analyses, we ran a first model included all participants and cycle phases to compare the pre-ovulatory and the luteal phase to menses, and a second model excluding menses to compare pre-ovulatory and luteal phase to each other. The interaction between hemisphere × cycle was non-significant and removed for all models. To assess whether cycle effects were attributable to estradiol or progesterone, models showing a significant cycle effect were rerun, replacing cycle phase by estradiol × progesterone values (e.g., eigenvariate ~ 1|PNr + session + estradiol × progesterone). A cutoff of 3 standard deviations from the mean was applied for the relational analyses to remove outliers in the hormonal values (2 in estradiol and 4 in progesterone). Both the dependent variables and hormone values were scaled prior to analyses to allow for interpretation of effect sizes based on standard deviations, similar to Cohen’s *d*. *p* values were FDR corrected for the number of ROIs. Data and scripts are openly available at https://webapps.ccns.sbg.ac.at/OpenData/. MR images are available upon request from the first author.

## Results

### Demographic data

For five participants, neither estradiol nor progesterone levels were in the expected pattern range across cycle phases, four participants had unusually low levels of progesterone in the luteal phase and nine participants did not show the expected pre-ovulatory estradiol peak. Accordingly, they were excluded from further analyses resulting in a total sample of 60 naturally cycling women (Table 3).

**Fig. 1:**
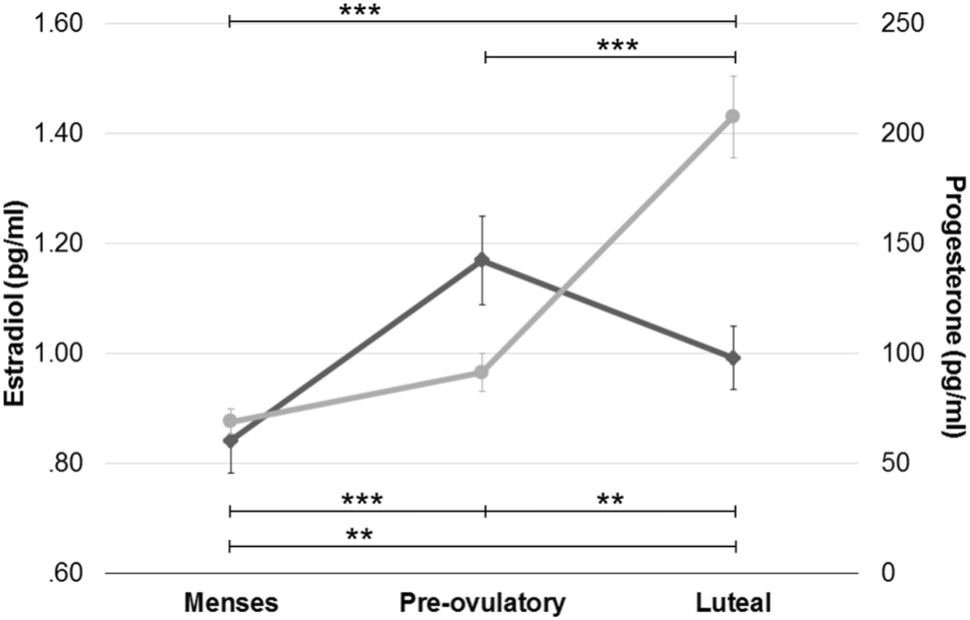
Hormone levels during each cycle phase: values are presented as mean $\pm$ standard error of the mean (M $\pm$ SEM). *Corresponds to *p* < 0.05, **corresponds to *p* < 0.01, and ***corresponds to *p* < 0.001

For the final sample and as expected, estradiol levels were significantly higher during the pre-ovulatory phase compared to menses (*b* = 0.62, SE_b_ = 0.10, *t*_(118)_ = 6.33, *p* < 0.001) and luteal phase (*b* = − 0.32, SE_b_ = 0.11, *t*_(59)_ = −  2.92, *p* < 0.01), as well as during the luteal phase compared to menses (*b* = 0.28, SE_b_ = 0.10, *t*_(118)_ = 2.91, *p* < 0.01) (Table 3). Progesterone levels were significantly higher during the luteal phase compared to menses (*b* = 1.23, SE_b_ = 0.12, *t*_(118)_ = 10.54, *p* < 0.001) and pre-ovulatory phase (*b* = 0.92, SE_b_ = 0.11, *t*_(59)_ = 8.17, *p* < 0.001), but did not differ between the pre-ovulatory phase and menses, as expected (*b* = 0.20, SE_b_ = 0.12, *t*_(118)_ = 1.70, *p* = 0.09) (Table 3, Fig. 1).

### Neuroimaging results

#### Group ICA

**Fig. 2:**
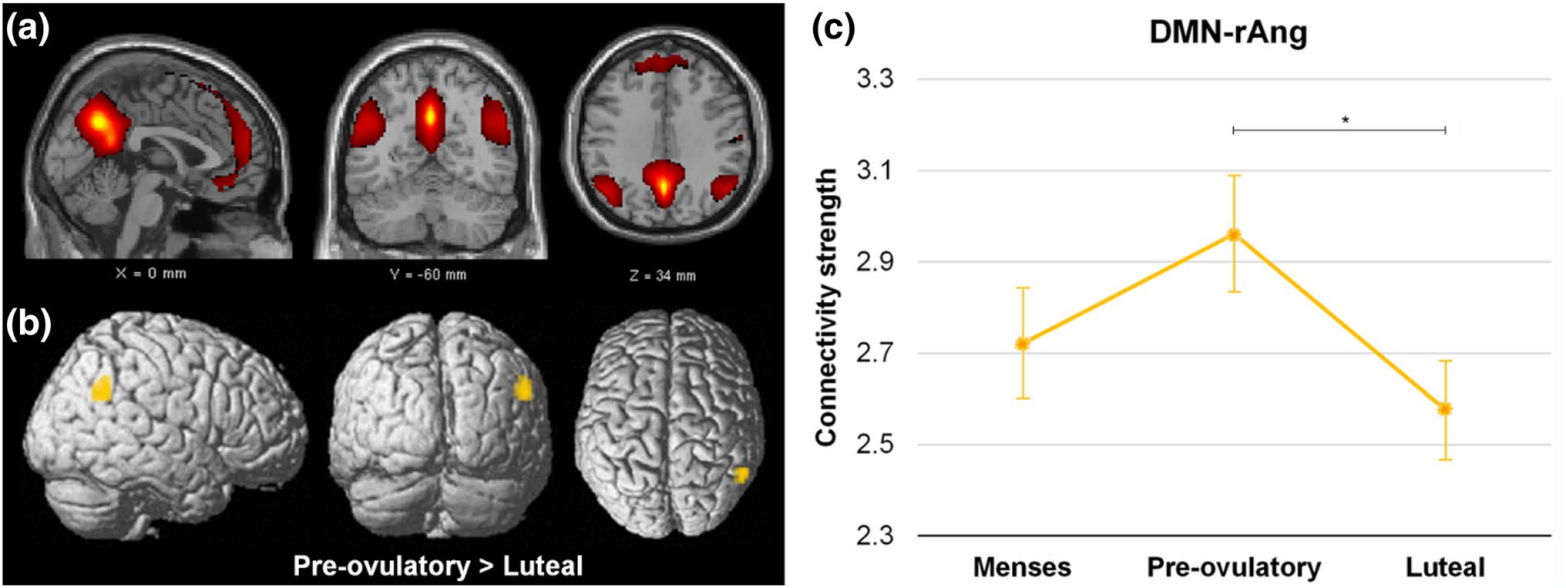
**a** Component 19 corresponds to ICN13 with a spatial correlation of 0.61, which was identified by Laird et al. (2011) as the DMN. **b**, **c** Menstrual cycle-dependent changes: connectivity was decreased during the luteal phase compared to the pre-ovulatory phase in the right angular gyrus [57–55 31] (in orange), and correlated to increased progesterone and decreased estradiol levels

Component 19 was the only component to show significant differences along the menstrual cycle. We identified this network as correspondent to ICN13 (*r* = 0.61), described as the DMN by Laird et al. (2011), and including medial prefrontal and posterior cingulate/precuneus areas (Fig. 2a, in red). Intrinsic connectivity in component 19 was decreased during the luteal phase compared to the pre-ovulatory phase within the right angular gyrus ([57–55 31], *T* = 4.48, *k* = 26 voxels, *p*_FWE_ = 0.026) (Fig. 2b, c, in orange).

Accordingly, the strength in connectivity was positively related to estradiol levels (*b* = 0.16, SE_b_ = 0.07, *t*_(110)_ = 2.37, *p* = 0.02) and inversely correlated to progesterone levels (*b* = − 0.13, *SE*_b_ = 0.05, *t*_(110)_ = − 2.72, *p* < 0.01), but no interaction effects were observed (*b* = − 0.01, SE_b_ = 0.04, *t*_(110)_ = − 0.35, *p* = 0.73).

#### ECM

**Fig. 3:**
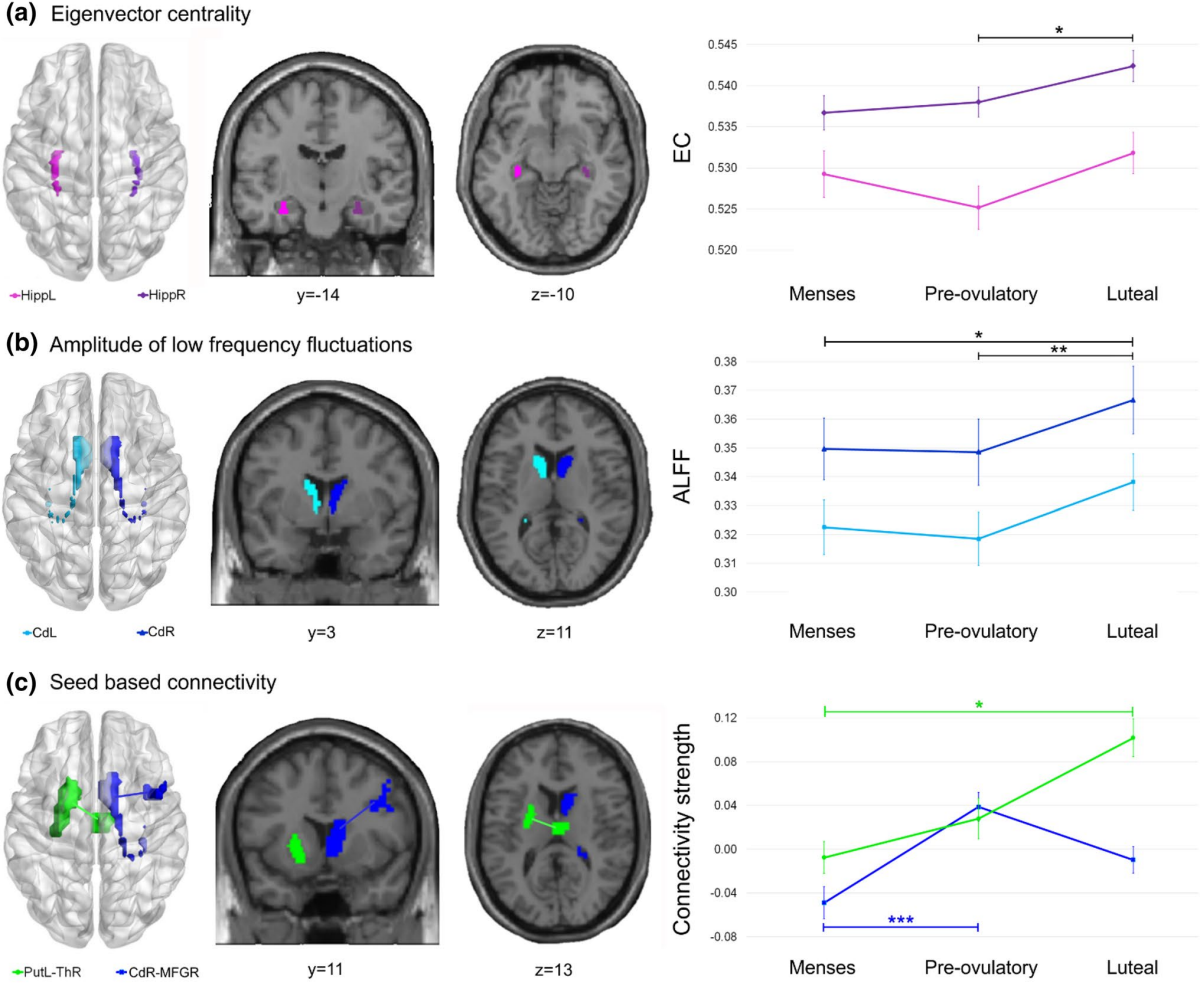
Areas found to be modulated by cycle phase organized by method: **a** Eigenvector centrality (EC) values increased during the luteal compared to pre-ovulatory phase in the hippocampus (in purple). **b** The amplitude of low-frequency fluctuations (ALFF) was significantly stronger during the luteal compared to the pre-ovulatory phase and menses in caudate (in blue) and related to decreased estradiol and increased progesterone levels. **c** For the seed-based analyses, functional connectivity increased from menses to the pre-ovulatory phase between the right caudate and the right MFG (in blue) and from menses to the luteal phase between the left putamen and the right thalamus (in green). *Corresponds to *p* < 0.05, **corresponds to *p* < 0.01, and ***corresponds to *p* = 0.001. Black significance bars apply to both hemispheres. *Hipp* hippocampus, *Cd* Caudate, *Put* putamen, *MFG* middle frontal gyrus, *Th* Thalamus, *r* prefix: right, *l* prefix: left

For hippocampus, EC was significantly higher in the right hemisphere during all cycle phases (*b* = 0.55, SE_b_ = 0.09, *t*_(296)_ = 5.94, *p*_FDR_ <0.001). Irrespective of hemisphere, we found an increase in hippocampal EC values during the luteal phase compared to the pre-ovulatory (*b* = 0.30, SE_b_ = 0.11, *t*_(177)_ = 2.71, *p*_FDR_ =0.02), but not to menses (*b* = 0.22, SE_b_ = 0.11, *t*_(296)_ = 1.96, *p*_FDR_ = 0.15) (Fig. 3a). No significant changes occurred during the pre-ovulatory phase compared to menses (*b* = − 0.07, SE_b_ = 0.11, *t*_(296)_ = −  0.63, *p*_FDR_ = 0.75). None of these changes were related to hormonal levels (all |*b*|< 0.06, all SE_b_ > 0.07, all |*t*|< 1.10, all *p* > 0.05).

We did not find any significant changes in EC across the menstrual cycle for bilateral caudate or bilateral putamen (all |*b*|< 0.11, all SE_b_ > 0.15, all |*t*|< 0.70, all *p* > 0.05).

#### ALFF

For the caudate, ALFF was significantly higher in the right hemisphere during all cycle phases (*b* = 0.35, SE_b_ = 0.07, *t*_(296)_ = 5.18, *p*_FDR_ < 0.001). Irrespective of hemisphere, caudate ALFF was significantly stronger during the luteal phase compared to the pre-ovulatory (*b* = 0.23, SE_b_ = 0.07, *t*_(177)_ = 3.15, *p*_FDR_ < 0.01), and menses (*b* = 0.20, SE_b_ = 0.08, *t*_(296)_ = 2.44, *p*_FDR_ = 0.045) (Fig. 3b). No significant changes occurred during the pre-ovulatory phase compared to menses (*b* = − 0.03, SE_b_ = 0.08, *t*_(296)_ = −  0.35, *p*_FDR_ = 0.72). Accordingly, ALFF in the caudate showed a negative relation to estradiol levels (*b* = − 0.23, SE_b_ = 0.06, *t*_(283)_ = −  3.83, *p* < 0.001), and a positive relation to progesterone levels (*b* = 0.12, SE_b_ = 0.04, *t*_(283)_ = 2.68, *p* < 0.01), but no interaction effects were observed (*b* = − 0.003, SE_b_ = 0.04, *t*_(283)_ = − 0.08, *p* = 0.93).

We did not find any significant changes in ALFF across the menstrual cycle for bilateral hippocampus or bilateral putamen (all |*b*|< 0.21, all SE_b_ > 0.12, all |*t*|< 1.80, all *p* > 0.05).

In the exploratory whole-brain analyses, we did not find significant changes across the menstrual cycle for EC or ALFF. No voxel survive uncorrected *p* < 0.001 and *p*_FWE_ < 0.05.

#### Seed-based connectivity analyses

To explore whether the connectivity patterns from these ROIs to the rest of the brain change along the menstrual cycle, seed-to-voxel connectivity maps of each ROI were estimated.

##### Hippocampus

For the hippocampus, overall positive connectivity was found to contralateral hippocampus, bilateral superior and middle frontal gyri, bilateral precuneus, bilateral orbitofrontal cortex, bilateral parahippocampal, left superior medial gyrus, left caudate, left amydala, ipsilateral parietal superior and inferior gyri, ipsilateral precentral and postcentral gyri, ACC, right fusiform, and cerebellum among others (Fig. 1a, b), supplementary material).

No significant changes were observed in the connectivity patterns of bilateral hippocampus along the menstrual cycle.

##### Caudate

Overall, bilateral caudate showed positive connectivity with ipsilateral superior and medial frontal gyri, anterior cingulate cortex (ACC), putamen and contralateral cerebellum and caudate. Left caudate further showed positive connectivity with ipsilateral middle and inferior frontal gyrus and contralateral fusiform gyrus (Fig. 1c, d), supplementary material).

Connectivity of the right caudate to the right middle frontal gyrus (MFG) increased significantly from menses to the pre-ovulatory phase ([39, 11, 31], *T* = 4.33, *k* = 66 voxels, *p*_FWE_ = 0.001) (Fig. 3c, in blue). Neither estradiol nor progesterone levels were related to these changes (all |*b*|< 0.11, all SE_b_ > 0.05, all |*t*|< 1.40, all *p* > 0.05).

Connectivity of the left caudate showed no changes along the menstrual cycle.

##### Putamen

Overall, bilateral putamen showed positive connectivity with contralateral putamen, bilateral precentral and contralateral postcentral gyri, bilateral supplementary motor area, bilateral frontal superior gyri, olfactory areas, bilateral temporal lobe, cerebellum and other basal ganglia structures (Fig. 1e, f), supplementary material).

Along the menstrual cycle connectivity from the left putamen to contralateral dorsomedial thalamus increased significantly during the luteal phase compared to menses ([3, − 13, 13], *T* = 4.66, *k* = 34 voxels, *p*_FWE_ = 0.024) (Fig. 3c, in green). This increase was positively related to estradiol levels (*b* = 0.18, SE_b_ = 0.08, *t*_(110)_ = 2.23, *p* = 0.03), but not progesterone levels (*b* = 0.05, SE_b_ = 0.08, *t*_(110)_ = 0.66, *p* = 0.51) or their interaction (*b* = − 0.01, SE_b_ = 0.06, *t*_(110)_ = − 0.20, *p* = 0.84). Connectivity of the right putamen showed no changes along the menstrual cycle.

## Discussion

**Fig. 4:**
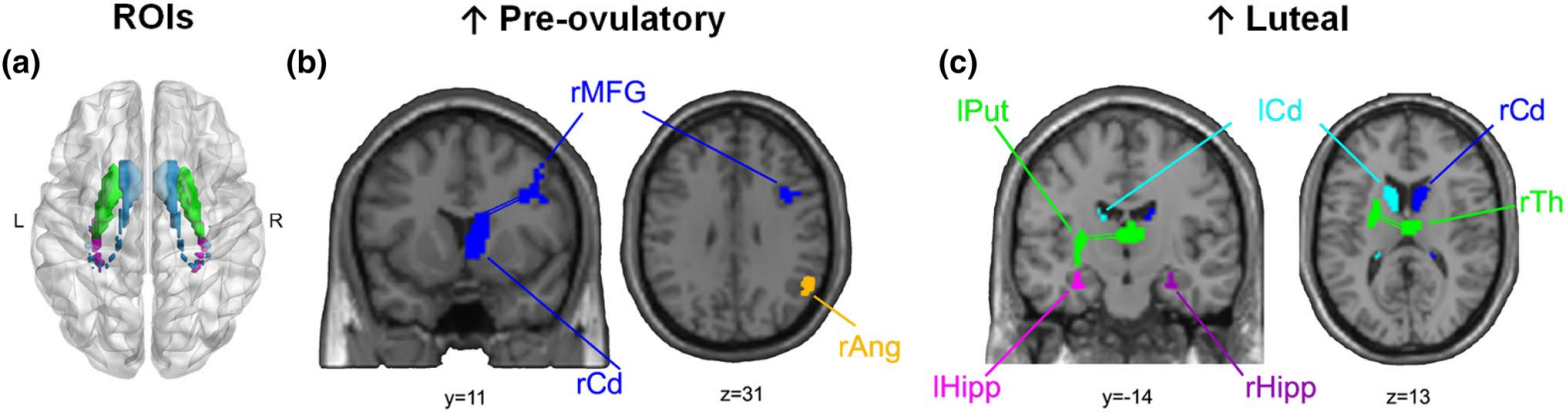
Overview of changes in RS-fMRI across the menstrual cycle. **a** Glass brain view of ROIs included in the analyses. Resting-state measures increased during **b** pre-ovulatory, and **c** luteal. *Ang* angular gyrus, *Hipp* hippocampus, *Cd* Caudate, *Put* putamen, *MFG* middle frontal gyrus, *Th* Thalamus, *r* prefix: right, *l* prefix: left

Over the recent years, menstrual cycle research in the neurosciences has considerably increased. The development of more sophisticated methods for brain imaging analyses has greatly contributed to our understanding of how endogenous sex hormone fluctuations affect the female brain. Nevertheless, literature characterizing women’s brain in each of the hormonal milieus is sparse and several inconsistent findings have been reported (Poromaa and Gingnell 2014). These discrepancies could be explained by the lack of systematic experimental designs and the heterogeneity in menstrual cycle monitoring across different studies. This is especially the case in RS analyses, although—in line with animal research—some areas consistently show changes across the different cycle phases. The aim of this study was to apply a multimodal analysis approach to characterize RS changes across the menstrual cycle within the same women. We focused on brain areas that previously emerged as key nodes of high functional relevance for whole-brain communication and targets of sex hormone effects. These ROIs included the hippocampus, putamen and caudate (Fig. 4).

The ROI-based approach revealed several interesting changes along the menstrual cycle. Centrality of the bilateral hippocampus increased during the luteal phase suggesting a stronger overall connectivity to other higher order hubs in this phase. While we expected centrality to be higher right before ovulation, during the mid-luteal phase estradiol and progesterone levels are higher compared to menses, and both hormones have been shown to drive neuroplasticity (for review, see Catenaccio et al. 2016). These results are in line with findings from Arélin et al. (2015), where the increased connectivity between hippocampus and sensorimotor cortex was related to increased progesterone levels. Alternatively, the increase in the hippocampus’ hierarchical position within the global network connectivity could reflect a compensatory mechanism for the drop in activation observed during the luteal phase (Pletzer et al. 2019).

As expected, the basal ganglia showed a significantly higher ALFF during the luteal phase but only for the caudate. Similarly, in a task-based design, we have recently observed an increased caudate activation irrespective of the task performed (Pletzer et al. 2019). Although the relationship between ALFF and structure is not yet clear, an increase in caudate GM has been related to impaired pain and emotion processing, which are also altered during the luteal phase (Catenaccio et al. 2016). Moreover, both increased activation and GM in the caudate have been related to impulsivity (Schneider et al. 2010; Tschernegg et al. 2015) and increased ALFF in the caudate nuclei has been related to major depression and bipolar disorder (Liu et al. 2012, 2014). Furthermore, in the present study, the connectivity between the right caudate and the ipsilateral MFG increased significantly during the pre-ovulatory phase and between left putamen and contralateral dorsomedial thalamus during the luteal phase. Putamen–thalamic coactivation has been consistently observed in human neuroimaging studies, both included in the fronto-striatal loops which regulate sensorimotor and executive functions (see metanalysis Postuma and Dagher 2006). A well-established measure of sensorimotor gating is the prepulse inhibition (PPI). PPI has been reported to change along the menstrual cycle, being increased during the follicular phase and reduced during the luteal phase (Swerdlow et al. 1997; Jovanovic et al. 2004; Bannbers et al. 2012). Moreover, dorsomedial thalamus is not only involved in sensorimotor regulation and the salience network, but has also been suggested to play a key role in cortico-cortical regulation of higher cognitive processes, such as learning and decision-making (Mitchell 2015). Overall, the increased caudate oscillatory activity and putamen–thalamic connectivity, with a decreased frontal regulation during the luteal phase, could underlie the vulnerability of this phase to affective disturbances such as premenstrual dysphoric disorder (PMDD). Accordingly, both impulsive behaviour and mood show a similar pattern of changes along the menstrual cycle as caudate ALFF and caudate MFG connectivity in the present study. Evidence suggests an estradiol-dependent pre-ovulatory reduction in impulsive symptoms (Diekhof 2015) and increased positive mood (Backstrom et al. 1983; Poromaa and Gingnell 2014; Toffoletto et al. 2014), but an increase in impulsive behaviour (Howard et al. 1988; Roberts et al. 2018) and decreased or fluctuating mood during the luteal phase (Poromaa and Gingnell 2014; Toffoletto et al. 2014).

Additionally, in the ICA analyses, we found changes along the menstrual cycle in the DMN, in line with two previous studies (Petersen et al. 2014; Weis et al. 2019). As reported in Petersen et al. (2014), we observed decreased intrinsic connectivity of the angular gyrus within the DMN during the luteal phase. RS-ICNs relate to previously observed task-based BOLD activation patterns and have been linked to different perceptual, emotional and cognitive functions (Biswal et al. 1995; Calhoun et al. 2008; Smith et al. 2009; Laird et al. 2011). Altered coherence within the DMN has been associated with atypical patterns in this network, related to altered introspective mental processes in disorders such as depression (Broyd et al. 2009). As previously suggested, this could affect introspectively oriented and self-referential mental activity throughout the menstrual cycle (Petersen et al. 2014) and relate to altered mood in the luteal cycle phase. However, the ICA approach has also yielded a number of inconsistent findings across studies.

The inconsistencies found in the literature could be partly attributable to small sample sizes, different methodological protocols and multiple analytical approaches that have been used. Furthermore, sex hormonal fluctuations along the menstrual cycle are short-lived and menstrual cycle effects usually yielded only small effect sizes. Relatedly, a majority of studies use analysis approaches at the whole-brain level. In the present study, as a secondary exploratory analysis, the whole-brain analyses on EC mapping and ALFF did not yield any significant results. It is plausible that, in combination with relatively small sample sizes, a whole-brain approach using current statistical methods is not sensitive enough to detect such subtle effects (Poldrack 2007; Eklund et al. 2016; Cremers et al. 2017). In fact, when comparing two cycle phases, the required effect size to survive a significance threshold of *p* = 0.000001 (which corresponds to FWE-correction at peak-level for 50.000 voxels) with 80% power and 60 subjects is *d* = 0.82. Therefore, whole-brain analyses would only be able to detect effects of this magnitude. Based on the present results, we conclude that the ROI-based approach appears particularly suitable for menstrual cycle designs.

Although RS-fMRI constitutes a valuable measurement of the brain’s intrinsic organization, we acknowledge that the interpretation of its results in terms of functional meaning should be cautious. Moreover, there are uncontrolled factors such as heart and respiration rate that could potentially affect the signal (Buckner et al. 2013). While they still may contribute, changes across menstrual cycle in these factors have been found non-significantly or quite inconsistently (Sato et al. 1995; Leicht et al. 2003; Peltonen et al. 2016), Thus, it is highly unlikely that they drive the signal changes that we observed. It is also worth noting that not in all cases we found a linear relationship between the salivary hormone levels and the RS measure. As has been argued before, this suggests that the changes cannot be completely explained in a monotonic manner or/and solely by the hormonal effect, excluding their interaction with multiple neurotransmitter systems (Petersen et al. 2014; Bayer et al. 2018). Finally, the present study remarks the importance of combining several analysis modes and integrate their results. For example, Syan et al. (2017) did not find significant differences across menstrual cycle with an ICA approach, but reported several effects related to hormonal fluctuations in the seed-based analyses (Syan et al. 2017). Each index and analysis assesses different RS measurements (for review, see Cole et al. 2010) and, therefore, there is not always a suitable corresponding proxy which can be compared across methodological approaches.

In summary, this is the first and largest longitudinal study that applies a multimodal analysis approach to investigate the influence of physiological ovarian hormonal modulation on RS activity and functional connectivity. We found effects in brain regions that are rich in steroid receptors, susceptible to show structural and functional changes across the menstrual cycle, and consistent with converging evidence from animal and human research. Specifically, during the pre-ovulatory phase, we observed stronger fronto-striatal connectivity in the right hemisphere. During the luteal phase, we found decreased intrinsic connectivity within the DMN, increased centrality for hippocampus, increased oscillatory activity for the caudate and stronger putamen–thalamic connectivity. The present results are in accordance with previous studies, both task-related and during resting state, describing increased subcortical connectivity, a higher reactivity of the salience network, and decreased coherence of the default mode network (Andreano et al. 2018). Those changes have been interpreted in terms of underlying changes in vigilance, executive processes, emotional, or sensorimotor regulation such as pain perception, already described during the luteal phase (Petersen et al. 2014; Arélin et al. 2015; Engman et al. 2016, 2018). Moreover, a triple network model of aberrant organization has been proposed to underlie several psychiatric and neurological disorders, composed by the central executive, the default mode and the salience network (Menon 2011), all of which are reported to vary in the present study. Therefore, we suggest that the fluctuations we observed in RS activity and functional connectivity reflect the ability of the healthy female brain to adapt to the acute physiological changes driven by ovarian hormones. The lack of such adaptive processes could make the brain more vulnerable to pathology, and the present results provide the framework for further investigation including women with menstrual cycle-associated disorders, such as PMDD or dysmenorrhea. Overall, while endogenous fluctuations are subtle, the implications for functional brain organization and, therefore, women’s health and well being emphasize the importance of understanding the female neural correlates as temporally dynamic.

## Electronic supplementary material

Below is the link to the electronic supplementary material.

Supplementary file1 (PDF 783 kb)
