# Supplementary material for: Human menstrual cycle variation in subcortical functional brain connectivity: a multimodal analysis approach
Source: Brain Struct Funct. 2020 Jan 1;225(2):591–605. doi: 10.1007/s00429-019-02019-z (PMC7046575; doi:10.1007/s00429-019-02019-z)

Article title:

Human menstrual cycle variation in subcortical functional brain connectivity - a multimodal analysis approach.

Journal:

Brain Structure and Function.

Author names:

Esmeralda Hidalgo-Lopez, Karsten Mueller, TiAnni Harris, Julia Sacher, Belinda Pletzer.

Affiliation and e-mail address of the corresponding author:

Department of Psychology and Centre for Cognitive Neuroscience, University of Salzburg, Hellbrunnerstr. 34, 5020 Salzburg, Austria

esmeralda.hidalgolopez@sbg.ac.at

ICN	Main structures implicated	Related function	Component	Spatial correlation (r)
Group 1: networks related to emotional and autonomic processes				
ICN1	Limbic and medial temporal areas	Emotion discrimination	17	0.28
ICN3	Bilateral basal ganglia and thalamus	Reward and interoception		0.15
ICN4	Executive control network including anterior insula and cingulate cortex	Linking cognition and emotion		0.27
ICN2	Subgenual ACC and OFC	Olfaction, gustation, and emotion	-	-
ICN5	Midbrain	Air, hunger	13	0.26
Group 2: networks related to motor and visuospatial integration and coordination				
ICN6	Middle and superior frontal gyri and SMA	Movement preparation	14	0.46
ICN7	Middle frontal gyri and posterior parietal lobules	Visuospatial attentional control	7	0.46
ICN8	Ventral precentral gyri, central sulci, postcentral gyri, superior and inferior cerebellum	Hand movements	6	0.49
ICN9	Superior parietal lobule (SPL)	Hand-eye coordination	3	0.57
Group 3: networks related to visual perception.				
ICN10	Temporal visual network	Visual perception	16	0.17
ICN11-12	Lateral and medial posterior occipital visual networks	Visual perception	5	0.70
Divergent networks.				
ICN13	Medial prefrontal and posterior cingulate/precuneus	Default mode network	19	0.61
ICN14	Cerebellum	Action and somesthesia	-	-
ICN15	Right-lateralized fronto-parietal network including right inferior frontal gyrus	Reasoning and working memory	11	0.46
ICN16	Primary auditory cortices	Audition	8	0.42
ICN17	Dorsal central cerebellar network, including dorsal precentral gyri, central sulci, postcentral gyri, superior and inferior cerebellum	Action, speech and diverse mouth movements	1	0.66
ICN18	Left-lateralized fronto-parietal network, including Broca's and Wernicke's areas	Verbal and memory tasks	10	0.41

Table 1. Correspondence between intrinsic connectivity networks (ICN) by Laird et al. (2011) and components extracted from group independent component analysis.

Figure 1: Resting state seed-based connectivity of the left (a) and right (b) hippocampus, left (c) and right (d) caudate, and left (e) and right (f) putamen in women. Axial slices, z coordinates depicted in mm.

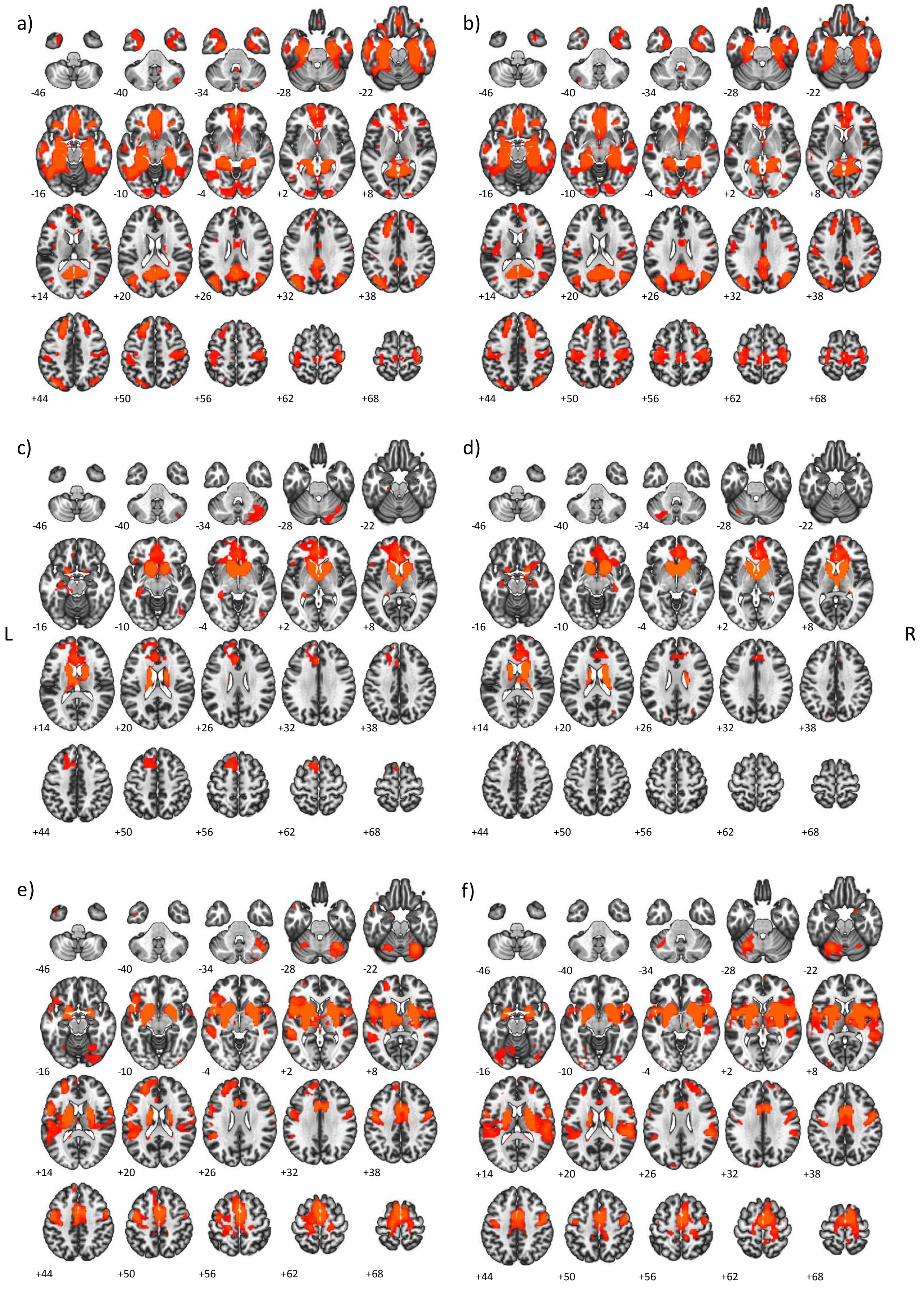

Supplement: Supplementary file 1 — Supplementary file1 (PDF 783 kb) [file 429_2019_2019_MOESM1_ESM.pdf]
